# Contrasting Carbon Allocation Strategies of Ring-Porous and Diffuse-Porous Species Converge Toward Similar Growth Responses to Drought

**DOI:** 10.3389/fpls.2021.760859

**Published:** 2021-12-16

**Authors:** Valentina Buttó, Mathilde Millan, Sergio Rossi, Sylvain Delagrange

**Affiliations:** ^1^Département des Sciences Naturelles, Institut des Sciences de la Forêt Tempérée, Université du Québec en Outaouais, Ripon, QC, Canada; ^2^Département des Sciences Fondamentales, Université du Québec à Chicoutimi, Chicoutimi, QC, Canada; ^3^Key Laboratory of Vegetation Restoration and Management of Degraded Ecosystems, South China Botanical Garden, Chinese Academy of Sciences, Guangzhou, China

**Keywords:** functional group, SPEI, primary growth, secondary growth, ramifications, stem elongation, hydraulic diameter, vessels

## Abstract

Extreme climatic events that are expected under global warming expose forest ecosystems to drought stress, which may affect the growth and productivity. We assessed intra-annual growth responses of trees to soil water content in species belonging to different functional groups of tree-ring porosity. We pose the hypothesis that species with contrasting carbon allocation strategies, which emerge from different relationships between wood traits and canopy architecture, display divergent growth responses to drought. We selected two diffuse-porous species (*Acer saccharum* and *Betula alleghaniensis*) and two ring-porous species (*Quercus rubra* and *Fraxinus americana*) from the mixed forest of Quebec (Canada). We measured anatomical wood traits and canopy architecture in eight individuals per species and assessed tree growth sensitivity to water balance during 2008–2017 using the standardized precipitation evapotranspiration index (SPEI). Stem elongation in diffuse-porous species mainly depended upon the total number of ramifications and hydraulic diameter of the tree-ring vessels. In ring-porous species, stem elongation mainly depended upon the productivity of the current year, i.e., number of vessels and basal area increment. Diffuse-porous and ring-porous species had similar responses to soil water balance. The effect of soil water balance on tree growth changed during the growing season. In April, decreasing soil temperature linked to wet conditions could explain the negative relationship between SPEI and tree growth. In late spring, greater water availability affected carbon partitioning, by promoting the formation of larger xylem vessels in both functional groups. Results suggest that timings and duration of drought events affect meristem growth and carbon allocation in both functional groups. Drought induces the formation of fewer xylem vessels in ring-porous species, and smaller xylem vessels in diffuse-porous species, the latter being also prone to a decline in stem elongation due to a reduced number of ramifications. Indeed, stem elongation of diffuse-porous species is influenced by environmental conditions of the previous year, which determine the total number of ramifications during the current year. Drought responses in different functional groups are thus characterized by different drivers, express contrasting levels of resistance or resilience, but finally result in an overall similar loss of productivity.

## Introduction

A reliable understanding of tree growth responses to environmental conditions is important in confronting the changes caused by the global warming. Strategies of carbon allocation in trees can be identified through the study of functional traits, i.e., morpho-physio-phenological features that affect all aspects of the life histories of living organisms (Violle et al., 2007). Plant physiological traits provide the basis for a conversation that is aimed at creating a harmonious modeling framework, integrating descriptive and experimental results into more advanced digital vegetation models (Yang et al., 2015).

Allometric scaling among the different plant organs drives primary and secondary growth toward convergent patterns to changing environmental conditions, leading to adjustments in biomass allocation and sapwood area (Petit et al., 2018). Yet, within the angiosperms, the presence of diverging wood types, e.g., ring-porous and diffuse-porous species, entails differing sensitivity to the environment, concurrently implying different degrees of dependency among functional traits in different organs (Palacio et al., 2011; García-González et al., 2016). Ring-porous and diffuse-porous species are thus expected to undergo contrasting growth responses to seasonal stresses, such as drought events. Given that these groups demonstrate divergent seasonal patterns in their developmental phases (Barbaroux and Bréda, 2002; Delpierre et al., 2016), they could manifest contrasting consequences in terms of resource partitioning between radial growth and stem elongation.

Bud break of ring-porous species is preceded by an earlier resumption of cambial growth, which enables the formation of large earlywood vessels in spring (von Allmen et al., 2015). These large vessels are efficient in conducting water, but prone to freeze–thaw embolisms and cavitation during drought events (Sperry et al., 2012; Kitin and Funada, 2016). Compared with ring-porous species, diffuse-porous species produce smaller vessels, but demonstrate a greater conductivity per stem area. They are also characterized by a lower vulnerability to cavitation (Taneda and Sperry, 2008). Nevertheless, during a drought event, diffuse-porous species of temperate biomes are demonstrably less efficient in controlling stomatal transpiration than are ring-porous species, resulting in declining water potentials and subsequent water transport (Bush et al., 2008). This response pattern is ascribable to an anisohydric behavior, which is achieved by maintaining both open stomata and high carbon assimilation rates, despite the risk of hydraulic failure (Martínez-Vilalta et al., 2014). According to their growth responses to drought events, plant survival can indeed be associated with isohydric or anisohydric regulation of water status, whereby these strategies depend upon the capacity to avoid hydraulic failure by closing the stomata or by tracking environmental variation by adjusting their leaf water potentials (McDowell et al., 2008). Compartmentalizing view of the anisohydric and isohydric spectrum does not explain sufficiently hydraulic strategies of trees, which implies mortality to drought happening through multiple mediated trait responses, not always directly linked to non-structural carbohydrate responses (Klein, 2014; Adams et al., 2017). Traits linked to non-structural carbohydrate responses, such as wood porosity, might thus not resolve by themselves the complexity of trees’ drought responses, which should be studied through multiple traits and physiological responses (Yi et al., 2017).

Like radial growth, the tree architecture is largely affected by wood type. Architecture is mainly defined by the number of ramifications and the number and degree of elongation of internodes, which results in sensitivity of response to environmental factors. Ring-porous species have the largest C pools, which are achieved through a greater capacity for storing carbon from the previous growing seasons (Barbaroux and Bréda, 2002; Palacio et al., 2011). This carbon pool, represented by the starch accumulated in form of reserves, is consumed to support earlywood growth, allowing for high growth rates independent of new photosynthates, and generating strong non-structural carbohydrate response fluctuations during the growing season (Michelot et al., 2012). In contrast to diffuse-porous species, ring-porous species do not change carbon allocation to growth over the lifespan of the trees. This response entails more stable lifetime allocation to shoot growth, which results in high water-use efficiency and a lowered amplitude of response to environmental variation compared to diffuse-porous species (Genet et al., 2009). Plant architecture can be affected by drought events, which inhibit the cell elongation and expansion, ultimately resulting in shorter internodes and smaller leaves (Song et al., 2021). Manipulation experiments confirm that water deficits involve an overall reduction in biomass accumulation by affecting plant carbon gain, which is linked to changes in stomatal conductance and light-capture efficiency (Valladares and Pugnaire, 1999; Rasheed and Delagrange, 2016). Studies demonstrate that canopy architecture is influenced by irrigation, which enhances biomass accumulation by increasing trunk volume and the number of branches (Devakumar et al., 1999).

To explore the response of species with divergent wood types, we must assess intra-annual processes that are involved in primary and secondary growth in an integrated manner. Indeed, a mutual correlation exists between primary and secondary growth. On the one hand, secondary growth depends upon non-structural carbon that results from the products of primary growth (Buttó et al., 2021). On the other hand, primary growth depends upon water conduction area, which in turn depends on secondary growth (Deslauriers et al., 2016). The functional traits of primary and secondary meristems, therefore, must be studied together to obtain a comprehensive framework of tree growth. In this study, we (i) investigated the relationship between primary and secondary growth in different wood-type species and (ii) assessed the sensitivity of their growth responses to interannual soil water content variation. We posit that wood type mirrors different carbon allocation strategies, resulting in contrasting growth responses to soil water content. We predicted that ring-porous species are more sensitive to short, but intense drought events because of their isohydric behavior, while diffuse-porous species are more sensitive to moderate, but long drought events because of their anisohydric behavior (Klein, 2014). We tested the hypothesis by analyzing primary and secondary growth during 2008–2017 on four sympatric species with contrasting wood types: American ash (*Fraxinus americana* L.) and red oak (*Quercus rubra* L.), which were ring-porous, and sugar maple (*Acer saccharum* Marsh.) and yellow birch (*Betula alleghaniensis* Britt.), which were diffuse-porous.

## Materials and Methods

### Study Area and Tree Selection

The study area is located within the Kenauk Nature Reserve, a large private property covering over 262 km^2^ in the region of Outaouais (QC), Canada. The study area has a mean elevation of 226 m asl and lies within the northern temperate zone, in the sugar maple–basswood western bioclimate domain where sugar maple grows alongside American basswood (*Tilia americana* L.), American ash, American hophornbeam (*Ostrya virginiana* [Mill.] K. Koch), and butternut (*Juglans cinerea* L.). This study involves four tree species: sugar maple, American ash, red oak, and yellow birch. Eight trees per species were selected and sampled in May 2018 among the dominant individuals that resulted from natural regeneration in a stand that was subjected to two strips of a clearcut in 1990 and three strips of a partial cut in 2005.

### Meteorological Data

Temperature and precipitation for 2008–2017 were extracted for the sampling area from the ERA5 monthly aggregate dataset of Google Earth Engine (Copernicus Climate Change Service [C3S], 2017; Gorelick et al., 2017), while standardized precipitation evapotranspiration index (SPEI) was extracted by the global SPEI dataset SPEI base v.2.6 (Beguería et al., 2014). Forests in cold and humid areas display drought responses over short time scales (3–5 months) (Vicente-Serrano et al., 2014). Accordingly, we selected SPEI_3_, which is calculated from the cumulative precipitation for periods of 3 months. Drought episodes were identified as the month with SPEI_3_ < −1 (Vanhellemont et al., 2019).

### Primary Growth Data

Stem elongation was measured for 2008–2017 as the interannual extensions of the annual shoot by detecting the cataphylls, i.e., scale leaves on the growth units of the main tree-axis (Barthélémy and Caraglio, 2007). The number of lateral ramifications was counted and characterized on each growing period, which were cross-validated for the attribution of the correct age and polycyclic-growth unit (Barthélémy and Caraglio, 2007). Lateral ramifications were classified as long, medium, and short, based on their length, number of internodes, and leaves (Gregory, 1980).

### Secondary Growth Data

A disk was cut from the stem of each tree at 1.30 m from the root-collar, and a radial section of 20 mm height × 5 mm width, which corresponded to stem disk half-diameter, was obtained from the most visible ray. From these rectangular samples, cross-sections of 51 tree cores were cut to about 10–15 μm thickness using a rotatory microtome (Leica, Heidelberg, Germany). The sections were stained with a solution of safranin (1% in distilled water), permanently fixed with BioMount mounting medium (Gärtner and Schweingruber, 2013), and photographed using a digital camera at 5× magnification. The digital images were analyzed using WinCELL™ (Regent Instruments Inc., Quebec, QC, Canada) for measuring ring-width (RW), vessel diameter, and vessel area. Hydraulic diameter of vessel (HDV) was computed, based up the study by Sperry et al. (1994):


$$\text{HDV}=\frac{\sum_{i=1}^{n}{\text{d}}^{5}}{\sum_{i=1}^{n}{\text{d}}^{4}}$$

where *d* is the diameter of the vessels from 1 to *n*, estimated from the measured area.

The annual basal area increment (BAI) was computed as:


$$\text{BAI}=\pi \,\left({\text{RW}}_{t}^{2}-{\text{RW}}_{t-1}^{2}\right)$$

where π*RW*^2^represents the basal area of the current (*t*) and previous (*t*-1) year.

Only individuals that were synchronized with the average tree-ring chronology for each species were retained for the following analyses. Tree-ring chronologies were considered well synchronized when Pearson’s correlation coefficients were >0.5.

### Relationship Between Growth and Environment in the Two Functional Groups

The most important predictors of stem elongation were obtained with random forest regressions, which were performed separately on the two functional groups of tree-ring porosity (i.e., diffuse-porous and ring-porous species). For this purpose, we tested SPEI_3_ and growth variables as predictors. A random forest is composed of a large number of decision trees that are obtained by random bootstrap sampling of the data. The random selection of a subset of predictors allows testing the capacity of each predictor to predict the variable with accuracy (Liaw and Wiener, 2002). Random forest and permutation feature importance computed to estimate the relative contribution of predictors in prediction accuracy were performed using the *RandomForest* package in R (Liaw and Wiener, 2002). Regression performance was evaluated by the root-mean-square error (RMSE) and by regressing observations against their predicted values with linear models.

General linear mixed models (GLMMs) were used to study the effects of tree-ring porosity, SPEI_3_ computed for each month (i.e., previous August–September and current April–July), and their interaction on growth. Growth variables were centered and normalized using the function *bestNormalize* of the *bestNormalize* package in R, which evaluates and applies the best normalization based upon the most efficient transformation (Peterson and Cavanaugh, 2019). GLMM was performed with the function *lmer* of the *lm4* package (Bates et al., 2015). Species and individual trees were retained as random effects; collinearity between variables was assessed through the calculation of variance inflation factors (VIFs) with the customized function *vif-func.r* (Beck, 2017). Only variables having VIF < 10 have been retained (Zuur et al., 2010). Normality and homoscedasticity of residuals were visually assessed. For each model, we computed the marginal and conditional *R*^2^ representing the contribution of variance explained by the fixed effects and by the sum of the fixed and random effects, respectively, with the *MuMIn* package in R (Barton, 2020). For the significant terms, the fixed effects with their interactions have been plotted by using predictions that were computed by means of the *ggeffects* package (Lüdecke, 2018).

We retained the drought events (SPEI_3_ < 1) between April and August to assess the effect of drought on tree growth, and we computed tree resilience to drought by identifying three characteristic periods that were defined as pre-drought, drought, and post-drought periods (Vanhellemont et al., 2019). A higher risk of mortality is associated with trees that have low resilience to drought (DeSoto et al., 2020). Due to its prolonged severity, we focused our attention on the drought event of 2012. We then considered 2009–2011 as the pre-drought period, 2012–2013 as the drought period, and 2014–2016 as the post-drought period. Following DeSoto et al.’s (2020) approach, we computed pre-drought and post-drought tree growth by averaging tree growth during 3 years prior to and following the drought period, respectively; growth patterns have been visually compared and discussed.

## Results

### Weather Conditions and Drought Events

**FIGURE 1:**
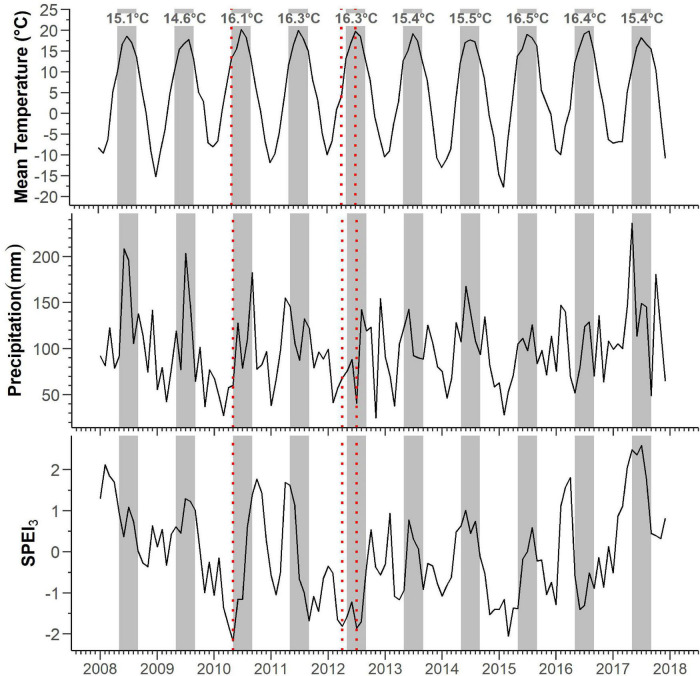
Patterns of monthly mean temperature, total precipitation, and SPEI_3_ 3-month windows. Red-dotted bars identify periods of severe drought (SPEI_3_ < -1) occurring during May 2010, April and July 2012. Gray bars and annotations mark the May–September periods and their respective mean temperatures.

The mean temperature of the study area was 5.1°C, with an annual precipitation of 1,189 mm, while the absolute maximum and minimum temperatures for 2008–2017 were 29.0 and -28.9°C, respectively (Supplementary Figure 1). Frost occurred mainly from October to May, although frost events were also likely in September (Supplementary Figure 1). During 2008–2017, the mean monthly maximum temperature was 20 °C, which was reached in July, while the mean monthly minimum temperature was -12°C, which was measured in January (Figure 1).

The mean maximum SPEI_3_ was 1.31, with peaks observed in April during 2011 (SPEI_3_ = 1.7) and 2016 (SPEI_3_ = 1.8), in February of 2008 (SPEI_3_ = 2.1) and 2013 (SPEI_3_ = 0.9), and in October during 2010 (SPEI_3_ = 1.8) and 2012 (SPEI_3_ = 0.5). Occasionally, the maximum SPEI was observed in June (2014, SPEI_3_ = 1.0), July (2009, SPEI_3_ = 1.3), and August (2015, SPEI_3_ = 0.6). The mean minimum SPEI_3_ was −1.46, with minima recorded in November for the years 2008 (SPEI_3_ = −0.4), 2009 (SPEI3 = −0.9), and 2014 (SPEI3 = −1.5); April of 2013 (SPEI_3_ = −1.2); June of 2016 (SPEI_3_ = −1.4); and July of 2012 (SPEI_3_ = −1.9). During the spring–summer period, we detected three events of severe drought (SPEI_3_ < −1) occurring in May 2010, April and May 2012, and March 2015.

### Primary and Secondary Growth

**FIGURE 2:**
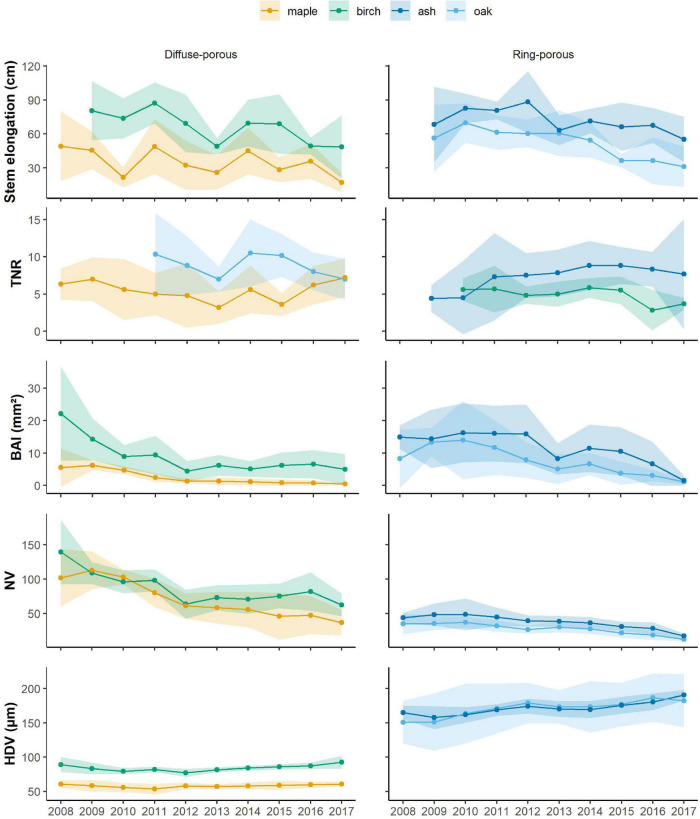
Average time series for the primary and secondary variables that were measured and computed, with light-colored bands representing standard deviations. Blue is ash and cyan is oak, while orange is birch and green is maple. TNR, total number of ramifications; BAI, basal area increment; NV, number of vessels; HDV, hydraulic diameter of vessels.

Except for the last 3 years of observation, diffuse-porous species, i.e., birch and maple, displayed synchronized stem elongation patterns (Figure 2). Compared with the other years, we observed that in 2012 and 2013, birch and maple experienced similar declines in stem elongation, losing, respectively, 21 and 34% of growth in 2012 and 30 and 20% of growth in 2013. Maple experienced two drops in stem elongation in 2010 (52%) and 2017 (53%). Birch showed a stem decline in 2016 (29%).

Ring-porous species, i.e., ash and oak, exhibited an average stem elongation of 60 and 80 cm, respectively, with slightly different temporal patterns (Figure 2). Ash stem elongation averaged 80 cm for the time period 2009–2012; oak stem elongation averaged 61 cm during 2009–2013, decreasing to 54.5 cm in 2014 and dropping to 34.5 during the last 3 years of observation (Figure 2). Ash exhibited the strongest decline in stem elongation in 2013 (28%). Oak displayed the strongest reduction in stem elongation in 2015, where growth reduced by 33%. In 2017, stem elongation in both ash and oak decreased by 18 and 15%, respectively.

Diffuse-porous species showed synchronized patterns in the number of total ramifications, except for the last 3 years (Figure 2). In both species, the minimum number of total ramifications was observed in 2013, when ramification decreased by 33% in maple and by 20% in birch (Figure 2). Within ring-porous species, oak displayed a larger interindividual variability in the total number of ramifications that were produced; when compared to ash, oak generally produced more ramifications starting from 2011, and the number of total ramifications averaged 8 over the observed period. More variability has been detected in the number of total ramifications that were produced by ash, which dropped by 48% in 2016 compared with the previous year.

For birch, BAI was exceptionally high in 2008 (22.1 mm^2^), progressively decreasing to a local minimum in 2012, where BAI decreased by 52% compared with the previous year. BAI slightly increased to 6.6 mm^2^ in 2016, but dropped to 5 mm^2^ in 2017 (Figure 2). For sugar maple, BAI averaged 5.5 mm^2^ during 2008–2010, dramatically decreasing by 48% in 2011 and by 34% in 2017. Within ring-porous species, ash had a greater basal area index, but the two time series were synchronized, showing constant growth until 2013, where the two curves declined together, displaying a growth reduction of 33% in oak and 48% in ash. Indeed, during 2008–2012, the average growth for ash and oak was, respectively, 15.5 and 11.8 mm^2^, dropping to 8.3 and 8.9 mm^2^ in 2013. Both species then experienced a growth decrease to reach a second local minimum in 2017, where growth declined by 77% in ash and 61% in oak (Figure 2).

The number of vessels for birch and maple averaged 142 and 105, respectively, slightly decreasing to a local minimum in 2012 for birch, when only 63 vessels had been produced. Vessel production progressively increased in birch reaching a local peak in 2016 (with 82 vessels) to decrease again in 2017 (62 vessels). In contrast, maple vessel production experienced a constant decline in number of vessels that were produced, which reached an absolute minimum in 2017, with a decline in vessel production by 40% when compared to the previous year. In 2012, both maple and birch experienced a decline in vessel production by 35 and 23% compared with the previous year. For ring-porous species, vessel production averaged 46 in ash and 35 in red oak during the period 2008–2011; in 2012, both species decreased by 12 and 17%, when 40 vessels and 26 vessels were observed. After 2012, the number of vessels in ash decreased until reaching a minimum of 17 vessels in 2017, while red oak vessels increased for two years (2013 and 2014), only to decrease again in 2016 and 2017 (Figure 2).

Hydraulic diameter of vessels showed a variation during the observed periods. Diffuse-porous species experienced a growth decline ranging by 6 and 1%, while growth release ranged between 1 and 8% when comparing the years of our time series. For ring-porous species, the hydraulic diameter growth decline ranged within 0.5 and 4%, while the growth release was between 2 and 8%. Between the diffuse-porous species, birch produced the largest vessels, with a hydraulic diameter of 83.4 μm during 2007–2011, which decreased by 6% in 2012 and progressively increased again to 92.6 μm in 2017. Maple displayed little variation in hydraulic diameter, which ranged from 53.3 μm (2011) to 60.5 μm (2017). On average, ash produced larger vessels than oak, displaying a hydraulic diameter of 163.5 μm during 2008–2011. Ash vessels’ hydraulic diameter increased to 174 μm in 2012, only to decrease again to 169 μm in 2014 and peak at 190 μm in 2017, displaying an increase by 6%. Oak produced vessels with a hydraulic diameter of 159.2 μm during 2008–2011, which became larger in 2012, where they measured 179 μm, and then decreased again in size until 2015. They peaked again in 2017, when diameters measured 182 μm (Figure 2).

### Relationship Between Apical, Radial Growth, and Weather Conditions

**FIGURE 3:**
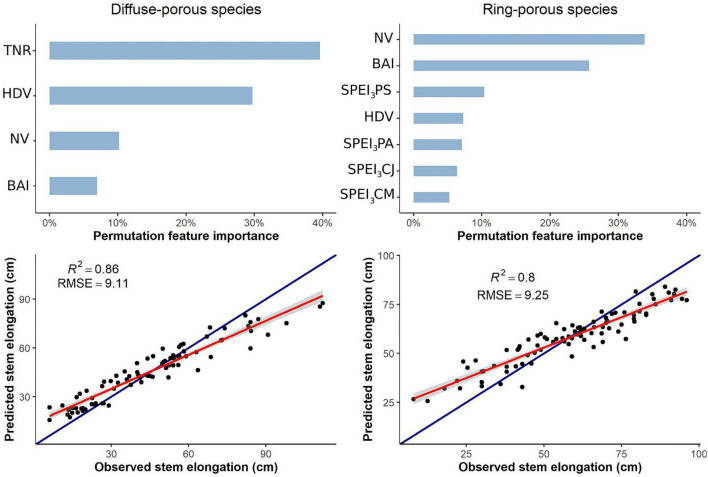
Random forest regressions for stem elongation, including secondary and primary growth variables. Only variables with a contribution ≥5% to variance explained are shown in the permutation feature importance plot. TNR, total number of ramifications; BAI, basal area increment; NV, number of vessels; HDV, hydraulic diameter of vessels; SPEI_3_ PA, SPEI_3_ of the previous August; SPEI_3_ PS, SPEI_3_ of the previous September; SPEI_3_ CM, SPEI_3_ of the current May; SPEI_3_ CJ, SPEI_3_ of the current July.

For diffuse-porous species, the random forest regression explained 38.79% of the variance, with an RMSE of 9.17. Predicted versus observed values were closely related (*R*^2^ = 0.86) (Figure 3). According to the permutation procedure, the most useful predictive variables for stem elongation were the total number of ramifications (36%), HDVs (26%), the number of vessels (9%), SPEI_3_ for August and BAI (6%), and SPEI_3_ of July (5%) The other anatomical and meteorological variables were not as important, ≤5% (Figure 3).

For ring-porous species, the random forest explained 15.83% of the variance. Predicted versus observed values were strongly related (*R*^2^ = 0.8), with an RMSE of 9.37. Under permutation, the number of vessels was revealed as the most useful predictive factor for stem elongation (36%), followed by BAI (25%), SPEI_3_ of May (8%), SPEI_3_ of July (7%), and SPEI_3_ of April and August (6%) (Figure 3). All other variables accounted for <5% of the variance.

**FIGURE 4:**
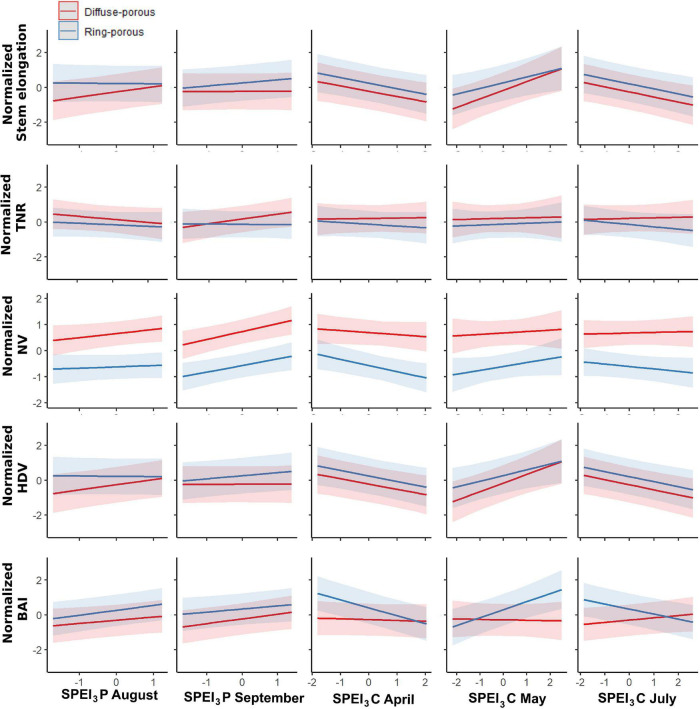
GLMM results for the two functional groups, red = Diffuse-porous, blue = Ring-porous. TNR, total number of ramifications; NV, number of vessels; HDV, hydraulic diameter of vessels; BAI, basal area increment; P, Previous year; C, Current year.

To circumvent the multicollinearity between SPEI_3_ of June and that of July, only the latter was integrated into the models. GLMM between the SPEI_3_ estimates for August and September of the previous year and March-to-August of the current year had a marginal *R*^2^ (*R*^2^_m_) that spanned a range from 0.08 when modeling the total number of ramifications to 0.6 when modeling the hydraulic diameter. Conditional *R*^2^ spanned (*R*^2^_c_) a range from 0.32 for the total number of ramifications to 0.88 for HDV (Table 1). Normalized stem elongation (*R*^2^_m_ = 0.16, *R*^2^_c_ = 0.60) was significantly affected by porosity, SPEI_3_ of April and their interaction, SPEI_3_ of August and September of the previous year, and SPEI_3_ in July. For the number of total ramifications, only the SPEI_3_ August × porosity and SPEI_3_ April × porosity interactions were significant, indicating an effect that was dependent on SPEI_3_ variation (Table 1). Under wet conditions, diffuse-porous species indeed display more ramifications than do ring-porous species (Figure 4). Normalized number of vessels (*R*^2^_m_ = 0.50; *R*^2^_c_ = 0.65) was significantly linked to the conditions of the previous August and September, and to SPEI_3_ of April, and its interaction with porosity (Table 1). In both functional groups, HDV (*R*^2^_m_ = 0.60; *R*^2^_c_ = 0.88) was significantly related to the variation in SPEI_3_ of the previous August and September, and to the variation in SPEI_3_ in the current April and May, without contribution of the interaction terms. Normalized BAI (*R*^2^_m_ = 0.20; *R*^2^_c_ = 0.65) mainly depended upon the SPEI_3_ variation in August and September of the previous year and SPEI_3_ variation from April to July of the current year (Table 1).

**TABLE 1:** Type I sums-of-squares (SS), *F*-statistics, and significance for the source of variation of the GLMM.

Source of variation	Stem elongation (cm) *R*^2^_m_ = 0.16; *R*^2^_c_ = 0.60		TNR *R*^2^_m_ = 0.08; *R*^2^_c_ = 0.32		NV *R*^2^_m_ = 0.50; *R*^2^_c_ = 0.65		HDV (μm) *R*^2^_m_ = 0.60; *R*^2^_c_ = 0.88		BAI (mm^2^) *R*^2^_m_ = 0.20; *R*^2^_c_ = 0.65	
					
	Type I SS	*F*-value (*P*)	Type I SS	*F*-value (*P*)	Type I SS	*F*-value (*P*)	Type I SS	*F*-value (*P*)	Type I SS	*F*-value (*P*)
POR	0.23	0.42	0.28	0.35	5.71	15.35	1.53	11.51	0.3504	0.76
SPEI_3_ P Aug	3.74	6.77 (*)	0.0196	0.02	8.51	22.87 (***)	0.54	4.10 (*)	13.48	29.18 (***)
SPEI_3_ P Sep	2.99	5.41 (*)	0.71	0.88	7.89	21.21 (***)	1.15	8.61 (**)	5.56	12.03 (***)
SPEI_3_ C Apr	2.43	4.40 (*)	0.20	0.25	3.97	10.68 (**)	1.21	9.13 (**)	4.34	9.40 (**)
SPEI_3_ C May	1.06	1.92	0.04	0.05	0.42	1.12	0.53	4.00 (*)	1.94	4.21 (*)
SPEI_3_ C Jul	10.65	19.27 (***)	0.26	0.33	0.24	0.65	0.09	0.66	0.77	1.67
SPEI_3_ P Aug × POR	1.53	2.76	4.56	5.69 (*)	1.40	3.77	0.20	1.52	0.12	0.27
SPEI_3_ P Sep × POR	0.48	0.87	0.05	0.06	0.02	0.05	0.01	0.10	0.00	0.01
SPEI_3_ C Apr × POR	2.68	4.85 (*)	5.13	6.41 (*)	1.93	5.20 (***)	0.00	0.02	3.69	7.99 (*)
SPEI_3_ C May × POR	0.65	1.18	0.07	0.09	0.03	0.07	0.13	1.00	0.01	0.03
SPEI_3_ C Jul × POR	0.001	0.001	2.14	2.67	0.49	1.31	0.49	3.71	6.08	13.15 (***)

*R^2^_m_, marginal R^2^ (fixed effect); R^2^_c_, conditional R-squared (random + fixed effect).*

*Species and individuals are the random effects.*

*SPEI_3_ of June has been excluded by VIF selection. TNR, total number of ramifications; NV, number of vessels; HDV, hydraulic diameter of vessels; BAI, basal area increment.*

*C denotes the current year, while P denotes the previous year and POR stands for the factor ring porosity.*

Asterisks represent the significance level: * P < 0.05; ** P < 0.01; *** P < 0.0001.

### Interannual Growth Trends

**FIGURE 5:**
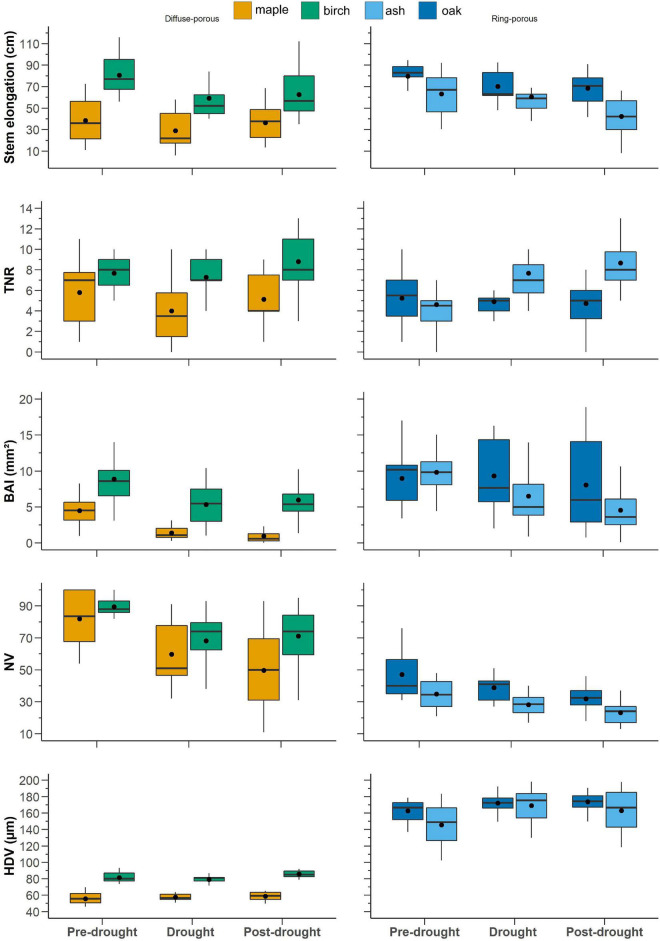
Trait responses to pre-drought period (2009–2010–2011), drought period (2012–2013), and post-drought period (2014–2015–2016). Black dots represent the average, continuous bars represent the median (50% quartile), lower and upper box limits represent the first and third quartiles, and vertical bars represent 1.5× the interquartile range. Blue is ash and cyan is red oak, while orange is birch and green is maple.

During stem elongation in the pre-drought period, diffuse-porous species exhibited an average stem elongation of 58.8 cm, which decreased to 45.5 cm during drought period and rebounded to 54 cm during the post-drought period (Figure 5). Diffuse-porous species produced, on average, 6 ramifications during pre-drought and drought periods and 10 ramifications during the post-drought period. The number of vessels was 100 during the pre-drought period and decreased to 64 for the drought period and the post-drought period. The HDVs were similar across the three periods, exhibiting values between 70 and 73 μm (Figure 5). On the one hand, ring-porous species showed a constant decline in stem elongation over the three time periods, with values ranging between 71 and 55 cm in the pre- and post-drought periods. On the other hand, the total number of branches constantly increased from 5 to 7 over the three periods (Figure 5). BAI was 11 mm^2^ during the pre-drought period, 8 mm^2^ during the drought period, and 7 mm^2^ during the post-drought period. The number of vessels showed a similar trend: 41 during the pre-drought period, 33 during the drought period, and 27 during the post-drought period (Figure 5). The decline in the number of vessels was associated with an increase in hydraulic diameter, which ranged from 162 μm during the pre-drought period to 177 μm during the post-drought period (Figure 5).

## Discussion

### Diffuse- and Ring-Porous Trees Display Different Carbon Allocation Strategies

Species with contrasting wood anatomy are characterized by different carbon allocations during the growing season because of their different requirements (Palacio et al., 2011). Despite different carbon allocation strategies, stem elongation of both functional groups is linked to xylem hydraulic traits. We observed that among xylem hydraulic traits, stem elongation is related to hydraulic diameter for diffuse-porous species and to the number of vessels for the ring-porous species. In ring-porous species, early conductivity can be performed by the small latewood vessels of the previous year, which can recover from winter embolism (Kitin and Funada, 2016). Nevertheless, the contribution of latewood vessels to overall intra-ring hydraulic conductivity is marginal (Dai et al., 2020). Indeed, most conduction of the xylem relies upon the larger earlywood vessels (Hacke and Sauter, 1996; Niu et al., 2017; Dai et al., 2020), which in turn rely upon the mobilization of the reserves that were accumulated during the previous growing season (Barbaroux and Bréda, 2002; Palacio et al., 2011). Consistent with our results, soil water balance at the end of the previous growing season is important in defining primary growth and earlywood vessel development in spring. The stronger influence of soil water balance of the previous year is likely related to the environmental conditions that are required for C accumulation before leaf senescence (Hartmann and Trumbore, 2016). While hydraulic diameter, which is mostly driven by allometric relationships (Carrer et al., 2014), remains relatively constant, the number of vessels displays greater variability. For ring-porous species, BAI, and consequently, xylem hydraulic efficiency, is more related to the number of vessels than to their diameter. A greater BAI is linked to an increase in the production of both wide and narrow vessels, with the first providing greater hydraulic efficiency during the periods of intensive growth, while the second is characterized by high hydraulic safety to cope with adverse climatic conditions (Kitin and Funada, 2016).

Diffuse-porous species can rely upon previous year tree-rings to conduct water upward in spring. As a consequence, they display larger sapwood area, which enables earlier bud break compared to ring-porous species (Gebauer et al., 2008). Indeed, the structure of their sapwood allows them to rapidly reverse winter embolism by refilling the xylem of previous tree-rings (Niu et al., 2017; Dai et al., 2020). Nevertheless, diffuse-porous species are limited by fewer and smaller vessels, which may explain their lower degree of stem elongation than ring-porous species. In diffuse-porous species, the total number of ramifications is the trait that is strongly predictive of stem elongation, suggesting that adequate environmental conditions during spring stimulate the initiation of lateral shoots; once established, the latter may contribute to the growth of the main stem. Evidence based upon observations made on maple and birch would suggest that short lateral shoots are specialized for light exploitation (i.e., carbon gain through photosynthesis), while long shoots are specialized for space exploration (Jarčuška and Milla, 2012; Taugourdeau et al., 2019). Similarly, by affecting sink strength for new growth, clipping treatments reduce tree-ring growth in diffuse-porous species, due to the loss of light exploitation shoot types (Palacio et al., 2011). Important C provisioning by lateral ramifications in diffuse-porous species may also explain the relative independence of their stem elongation in relation to environmental conditions that were experienced in the previous growing season and, thus, on carbon that was stored relative to ring-porous species (Barbaroux and Bréda, 2002; von Allmen et al., 2015). Moreover, C dating evidence demonstrates that stored C used for spring growth in sugar maple is not solely related to reserves that were accumulated in the previous year, but for longer periods. Indeed, these may span 3–5 years, thereby buffering the effects of low and high C storage years (Muhr et al., 2016).

### Intra-Annual Tree Growth Responses to Soil Water Balance

Our results point to a time and process dependency of growth responses to a variation in soil water balance, which might underlie differing seasonal sensitivity of the two functional groups to drought events. On the one hand, April (reactivation of xylem growth) and July (maturation of vessels) of the growing season seem to be important for basal increment of ring-porous species. On the other hand, August of the previous growing season, together with current April conditions, would seem to be critical in the establishment of lateral shoots. Since bud formation occurs late in the growing season (Barthélémy and Caraglio, 2007), the importance of soil water balance during August is surely related to these critical phases of ontogenesis (Buissart et al., 2018), while in April soil conditions are related to dormancy or the initiation of buds. In both functional groups, these intra-annual differences clearly confirm contrasting developmental phenologies, which still remain to be explored in depth. Nevertheless, these different specific processes occur during the same period of time, making the conditions of both previous and current year important for the two functional groups, although for contrasting reasons.

According to von Allmen et al. (2015), we observed that species belonging to different functional groups converge toward similar water usage, by taking advantage of different situations. Diffuse-porous species have a higher water transport capacity, but require more water for their growth. Ring-porous species are able to limit water consumption due to their efficiency in terms of water use (von Allmen et al., 2015). However, calibration issues with the heat-dissipation method might result in an underestimation of total sap flow in ring-porous species, while single-point sap flow measurements could neglect the contribution of the radial sap flow profiles within the inner xylem (Poyatos et al., 2007; Bush et al., 2010; Yi et al., 2017). Taken together, these methodological issues might artificially increase the observed gap in sap flow variation between diffuse-porous and ring-porous species during periods of high transpiration demand, even though the heat ratio method sensors are widespread tools that have been continuously improved since their establishment (Poyatos et al., 2007; Bush et al., 2010).

Diffuse-porous and ring-porous species cope with hydraulic recovery after winter embolism mainly by either refilling of existing rings *vs*. new ring formation from reserves, but eventually converge on a similar level of hydraulic efficiency during the growing season (Niu et al., 2017). In our study, despite belonging to different functional groups, seasonal trajectories of functional traits converge on similar responses to soil water balance. Dry conditions at the end of the previous growing season generally result in lower primary and secondary growth during the current growing season for both functional groups. The number of vessels and HDVs are the most sensitive traits for diffuse-porous and ring-porous species, respectively. Similar patterns of carbon allocation in response to drought have also been detected in Mediterranean evergreen (diffuse-porous) and deciduous (ring-porous) oaks. Corcuera et al. (2004a,b) observed that deciduous oaks responded to drought by decreasing RW, i.e., cell production, while evergreen oaks had narrow vessels. However, compared with evergreen oaks, deciduous oaks might be more affected by drought stress, since it resulted in the lack of latewood, where vessels display greater water-use efficiencies than earlywood vessels (Corcuera et al., 2004a). Under severe and repeated drought, the higher production of narrow rings featuring wide earlywood vessels and a low or lacking proportion of latewood expose Mediterranean ring-porous oaks to cavitation risks, potentially leading to an extensive dieback (Corcuera et al., 2004a). These pieces of evidence of shared strategies between the same functional groups growing in temperate and Mediterranean environments suggest that the hydraulic adjustments in response to water stress might be closely associated with their carbon allocation strategies, enabling these relationships to be generalized to other environments.

Lower plant water potential negatively affects carbohydrate mobilization and transport, ultimately resulting in reduced resource availability (Sala et al., 2010). Similarly, wet spring conditions (generally associated with cloudier sky and colder temperatures) could negatively affect soil thermal properties and meristem activity, which may explain the negative relationship between growth and soil water content in April. We raise the hypothesis that during spring, abundant winter snowfall can maintain the cold soil, thereby preventing growth reactivation of the roots (Rossi et al., 2011) and reducing the duration of the shoot elongation phase, eventually resulting in unfavorable conditions for tree growth. During late spring and summer, soil water content affects tree growth responses by modulating carbon partitioning (Deslauriers et al., 2016). Within plant organs, regardless of functional group, cell enlargement and cell division are both hydraulic-controlled processes that occur in parallel, but respond to water deficit in rather independent ways, while still affecting one another (Tardieu et al., 2011; Körner, 2015). Water absorption for tissue expansion may decrease soil water potential, which in turn may reduce stomatal conductance and carbon assimilation (Tardieu et al., 2011). This system of loose feedbacks between cell division and cell expansion might be the basis for delay between vessel number and hydraulic diameter variation in response to soil water content. Among conspecific individuals, nutrient, carbon, and water uptake is enhanced by root grafts, the development of which is triggered by trees’ proximity, thus constituting an element to be considered by managers (Gaspard and DesRochers, 2020). As a consequence of natural root grafting, close individuals display coupled responses to soil water content variation, adding further complexity to our understanding of drought responses in tree communities (Bader and Leuzinger, 2019).

### Tree Growth Responses to Identified Drought

During the study period, we detected three drought events: The first occurred during May 2010, while the others occurred in spring and summer 2012. In our chronologies, the effect of the drought events of May 2010 is barely noticeable, while the long-lasting drought events in 2012 were manifested in synchronous responses between functional groups, resulting in an overall loss of growth for both years 2012 and 2013. In diffuse-porous species, BAI and the number of vessels decreased in 2012, while stem elongation and the number of total ramifications decreased slightly in 2012 and more strongly in the following year, i.e., 2013. The number of shoots and lateral branches is predetermined and depends upon carbon storage of the previous year (Fournier et al., 2020). We observed that the number of total ramifications strongly depends upon SPEI_3_ variation during the previous year, especially in diffuse-porous species. In July, less carbon to sustain shoot growth results in a decreasing number of shoots, decreasing carbon assimilation for the following year. Apart from 2013, diffuse-porous species experienced partial recovery of tree growth, while ring-porous species experienced a general decline in BAI, but they maintained the similar hydraulic diameter among years. Such a result is consistent with measurements that are aimed at discriminating carbon-use strategies of ring-porous and diffuse-porous species (Barbaroux and Bréda, 2002; Palacio et al., 2011). By relying upon carbon stored during the previous growing seasons, ring-porous species could be more sensitive to repeated and long-lasting summer drought, despite their higher water-use efficiency, and by closing their stomata, being more prone to carbon limitation than diffuse-porous species (Levanič et al., 2011; von Allmen et al., 2015). In our study, droughts that occurred locally over the last decade did not allow us to test the importance of repeated seasonal (spring or summer) droughts, which in turn may highlight contrasting long-term responses between functional groups because of their contrasting phenologies.

By analyzing the effect of drought on different functional groups, Vanhellemont et al. (2019) had indeed demonstrated that ring-porous species were affected by recurring dry springs, while diffuse-porous species were more strongly affected by recurring summer droughts. The fitness of ring-porous or diffuse-porous species in a specific study area would then depend upon the future timing, duration, severity, and frequency of drought events. At ecosystem scale, disclosing the drivers of drought responses vis-à-vis climate change is compulsive and extremely dependent on our understanding of the plant physiological functioning, which demand for holistic studies encompassing both the belowground and the aboveground compartments of a tree (Abrams and Nowacki, 2016; Rodriguez-Dominguez and Brodribb, 2020). Emerging pieces of evidence point indeed to the dominant role of the rhizosphere in driving the hydraulic redistribution at individual level (Johnson et al., 2018; Rodriguez-Dominguez and Brodribb, 2020). Belowground conductance patterns have recently demonstrated to be good predictors of drought responses, pointing to losses in roots’ hydraulic conductivity and occurrence of shallow roots as the main drivers of species mortality (Johnson et al., 2018). It has been observed that root anatomy also plays a key role in setting hydraulic conductivity, which, in thin roots, is inversely related to the root length and the width of the root cortex (Rieger and Litvin, 1999).

## Conclusion

In this study, we investigated how the relationships between primary and secondary growth change among four sympatric species and with soil water content. We observed similar responses between ring-porous and diffuse-porous species to variations in soil water content. Results showed differences in carbon allocation strategies among the different functional groups, implying different trade-offs between primary and secondary growth. A strong connection links plant water status and carbon balance, especially in those species with a strong capacity for controlling stomatal closure (McDowell et al., 2008; Hartmann and Trumbore, 2016). Consequently, a drought event will trigger cascading negative feedback loops between primary and secondary growth. The two functional groups seem to show similar overall growth responses to drought, although these are based on different seasonal and physiological mechanisms. Studies integrating multiple plant traits are needed to unravel the fine-tuning of the mechanisms involved in drought responses at the individual level. Ring-porous and diffuse-porous species manifested common patterns that might enable management strategies to deal with the impacts of climate change while preserving their economic and ecologic values.

## Data Availability Statement

The raw data supporting the conclusions of this article will be made available by the authors, without undue reservation.

## Author Contributions

SD conceived and planned the experimental design. SD and MM carried out the sampling activities and performed the data collection. VB analyzed the data and wrote the first draft of the manuscript. SD and SR provided critical feedback to the manuscript. All authors commented and approved the final version of the manuscript.

## Conflict of Interest

The authors declare that the research was conducted in the absence of any commercial or financial relationships that could be construed as a potential conflict of interest.

## Publisher’s Note

All claims expressed in this article are solely those of the authors and do not necessarily represent those of their affiliated organizations, or those of the publisher, the editors and the reviewers. Any product that may be evaluated in this article, or claim that may be made by its manufacturer, is not guaranteed or endorsed by the publisher.
