# Supplementary material for: Contrasting Carbon Allocation Strategies of Ring-Porous and Diffuse-Porous Species Converge Toward Similar Growth Responses to Drought
Source: Front Plant Sci. 2021 Dec 16;12:760859. doi: 10.3389/fpls.2021.760859 (PMC8716880; doi:10.3389/fpls.2021.760859)
Supplement: Supplementary file 1 [file Data_Sheet_1.pdf]

Supplementary Figures

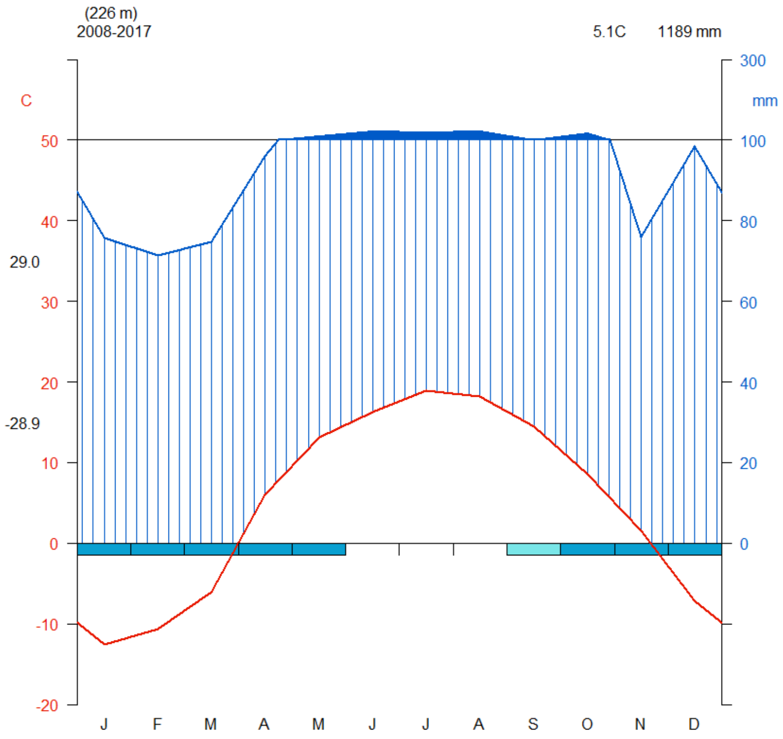

Supplementary Figure 1: Walter & Leith climatic diagram at the coordinates of the study area. Temperature and precipitation for 2008-2017 were extracted from the ERA5 monthly aggregate dataset of Google Earth Engine (Copernicus Climate Change Service [C3S], 2017; Gorelick et al., 2017). Beginning at the top left: elevation of the location, time span (2008-2017), mean temperature (5.1 °C) and total precipitation in mm. The red continuous line represents mean monthly temperature variation, with the absolute maximum and minimum daily average temperatures on labeled in black on the right axes. Vertical blue bars represent monthly precipitation in mm reported on the left axes. Blue and sky blue horizontal bars indicate frost period and likely frost period. A wet period is recorded when precipitation exceeds 100 mm and is indicated by an area filled in blue. Walter&Lieth climatic diagram was produced using the Climatol package (Guijarro, 2019).

References

Guijarro, J. A. (2019). climatol: Climate Tools (Series Homogenization and Derived Products). Available at: https://cran.r-project.org/package=climatol.
